# Phage vB_AbaM_MU1 for biocontrol of carbapenem-resistant *Acinetobacter baumannii* (CRAB) isolated from wound infection

**DOI:** 10.1186/s12985-026-03066-9

**Published:** 2026-02-06

**Authors:** Hadeer Sabry, Mai M. Zafer, Mohamed Abdelmoteleb, Ayat M. Hassan, Adel A. El-Morsi

**Affiliations:** 1 Botany Department, Faculty of Science, Mansoura University, Mansoura, Egypt; 2 Department of Microbiology and Immunology, Faculty of Pharmacy, Ahram Canadian University, Cairo, Egypt

**Keywords:** *A*. *baumannii*, Bacteriophage therapy, CRAB, Phage characterization, vB_AbaM_MU1

## Abstract

**Background:**

Carbapenem-resistant *Acinetobacter baumannii* (CRAB) continues to pose significant public health in clinical settings due to its remarkable genomic plasticity and resistance to available therapeutic drugs, including carbapenems. Bacteriophage has emerged as an optimistic solution capable of addressing such drug resistance dilemma. This study represents a comprehensive characterization of a novel *Acinetobacter* phage with potential application against CRAB-associated wound infections.

**Methods:**

Sewage sample was obtained, processed, and enriched with *A. baumannii* M13 phage(s) for the purpose of phages’ isolation. The isolated phage was examined using transmission electron microscope (TEM) and identified in terms of host range and efficiency of plating through spot test and plaque assay, respectively. Phage stability was screened following thermal, pH and ethanol assays. Replication kinetics were investigated through adsorption and single step growth curve. Furthermore, the in-vitro antibacterial potential was verified through measuring the optical density of the treated M13 culture at different Multiplicity of infections (MOIs) over 6 h shaking incubation. This is in tandem with preliminary screening of the vB_AbaM_MU1 safety through genomic and phylogenetic analysis of the isolated phage.

**Results:**

A novel lytic *Acinetobacter* phage vB_AbaM_MU1 was isolated and categorized as T4-like Myovirus with genomic size 167.200 bp, which was classified into the family *Straboviridae* in class *Caudoviricetes*, based on morphological and genomic analyses. It showed lytic efficiency against 9/17 CRAB strains. Infectivity and structural integrity revealed thermal stability up to 60℃, pH tolerance within pH range (3–11), sensitivity to different EtOH concentrations (10%, 50%, 75%, and 95%). In addition, vB_AbaM_MU1 displayed distinctive infection kinetics with 6 min adsorption, short latent (over 30 min), and high bursting (326 PFU/infected cell). The in-vitro bacteriolytic infectivity revealed robust and steady antibacterial action at MOI of 1 and above.

**Conclusion:**

These findings provide a strong, well-justified foundation for considering vB_AbaM_MU1 phage as successful candidate for phage therapy in treating CRAB- induced wound infections.

**Supplementary Information:**

The online version contains supplementary material available at 10.1186/s12985-026-03066-9.

## Introduction

*Acinetobacter baumannii* is a Gram-negative opportunistic bacterial pathogen naturally found in soil and aquatic environments [1, 2]. Despite being accepted as a typical component of human skin flora, its ability to survive under harsh conditions and persistence on surfaces combined with selective pressures in hospital settings, has made it a frequent cause of healthcare associated infections, particularly in critically-ill patients at intensive care units (ICUs) [3, 4]. This bacterium is known to form biofilm on medical devices including ventilators, urinary catheters, and central venous catheters, facilitating colonization and systemic spread [5]. Infections caused by *A. baumannii* include pneumonia, bloodstream infections, wound infections, meningitis, septicemia and urinary tract infections and associated with high rates of mortality [6, 7].

The emergence of multidrug-resistant (MDR) *A. baumannii* poses a clinical challenge as nearly all clinical isolates exhibit a broad resistance to multiple antibiotics, and disinfectants. This has been largely driven by extensive antibiotic use, especially β-lactams [8]. Carbapenems have lost efficacy after previous potency due to overuse, leading to carbapenem-resistant *A. baumannii* (CRAB). CRAB is associated with high morbidity and mortality, with resistance rates exceeding 90% in some regions and mortality from hospital-acquired pneumonia or bloodstream infections up to 60% [9].

*A. baumannii* is ranked in the highest quartile of the 2024 WHO bacterial priority pathogens list due to its high burden and health-care costs [10, 11]. In Egypt, MDR *A. baumannii* shows mortality rates up to 53.3% and carbapenem resistance of 26.6–100% among clinical isolates [12, 13].

Bacteriophages, viruses that specifically infect and replicate within bacteria, discovered over 100 years ago, offer a promising therapeutic solution. Lytic phages which efficiently kill their bacterial hosts are suitable for therapeutic consideration, whereas temperate phages integrate their respective genomes into the bacterial chromosome and may become dormant [14, 15].

Phages are highly host-specific, do not harm beneficial bacteria or human cells, and are naturally cleared from the body after their action [16]. They are abundant in environments such as wastewater, animal feces, and clinical samples and exhibit promising clinical viability, anti-inflammatory and immune modulation effects [17–20]. Several studies have demonstrated the efficacy of phages against CRAB infections, offering a viable solution where common antibiotics fail [21–24].

Genomic advances have accelerated the understanding of global viral diversity, enabled detailed characterization, classification, and safety assessment of phages, revealing evolutionary relationships [25–28]. This study reports the isolation, in vitro, and genomic characterization of vB_AbaM_MU1, a novel lytic phage targeting clinical CRAB isolates, providing insight into its potential antimicrobial applications.

## Materials and methods

### *Acinetobacter baumannii* strains

Eighteen CRAB clinical isolates were obtained from the Department of Microbiology and Immunology, Faculty of Pharmacy, Ahram Canadian University, Cairo, Egypt Table S1. These isolates were originally collected at the Clinical Pathology Laboratory of Kasr Al-Ainy University Hospital from a variety of clinical specimens including endotracheal tube aspirates, wound swabs, blood, sputum, pleural fluid and urine. All isolates were identified by VITEK^®^ 2 and subsequently confirmed by whole-genome sequencing (WGS), then submitted into the NCBI GenBank under BioProject No. PRJNA690827 [29]. *Acinetobacter baumannii* M13 isolate recovered from a wound infection and exhibiting the extensive drug resistant profile was selected as the host strain (BioSample No. SAMN17266003).

### Antibiotic susceptibility and growth conditions

The host *A. baumannii* strain SAMN17266003 (M13 strain), retrieved from glycerol stock at −20 °C, was streaked over MacConkey agar medium (HiMedia, India) and incubated overnight at 37 °C. Susceptibility to a variety of antibiotics classes was inferred by Kirby–Bauer disc diffusion test [30], including Cephems, *β*-Lactam combination agents, Carbapenems, Aminoglycosides, Tetracyclines and Fluoroquinolones. Susceptibility results were interpreted according to the Clinical and Laboratory Standards Institute (CLSI) guidelines [31]. For phage isolation, M13 was sub-cultured (CFU = 10^8^; OD_600_ = 0.25) over nutrient broth medium (0.6% Peptone, 0.5% NaCL, 2% yeast extract, 1% beef extract (w/v), and used for the subsequent work.

### Phage isolation and detection

Sewage sample was immediately manipulated after collection from Mansoura city, Egypt in early Autumn 2022 by centrifugation at 2000 × g for 30 min, followed by filtration using 0.22 μm syringe porous filter, and storage at 4℃ for next steps. For enrichment step, five ml post-treated sewage was mixed with 500 µl overnight culture of *A. baumannii* M13 cultured in 20 ml sterile nutrient broth medium, and followed by incubation at 37℃ overnight with shaking at 180 rpm [32].

Subsequently, the enriched sample was centrifuged, filtered, and screened for any lytic activity using spot-overlayer technique and plaques assay [33]. Briefly, 100 µl of *A. baumannii M13* strain was mixed with 5 ml soft agar (0.8% w/v agar), poured over nutrient agar plate (1.5% w/v agar). Then, 10 µl of filtered supernatant was spotted in triplicate over soft agar layer, and incubated at 37 ℃ for 24 h. The resulted spots were collected using 5 ml SM buffer, incubated at 4 ℃ for 1 h, centrifuged at 2000 × g for 20 min, filtered, and proceeded for plaque assay by serially diluting of filtrate in 900 µl SM buffer. Furthermore, 100 µl from each dilution was mixed with 100 µl *A. baumannii* M13 culture into 5 ml soft agar, and poured over solidified nutrient agar plates. Two clear-plaquing phages after incubation were detected against M13 strain.

### Phage purification and propagation

For purification of phage, each single plaque was individually picked by micropipette tip and suspended into 100 µl SM buffer [10 mM Tris HCl, 10 mM MgSO4, and 100 mM NaCl, pH 7.5]. Plaque assay was conducted by serial dilution till 10^− 10^, and the higher dilutions were plated at least three times till obtaining single plaque for each phage, before storage in SM buffer (pH 7.5) [33, 34]. Phage vB_AbaM_MU1 was further selected according to its host range analysis.

Phage stocks with high titer were prepared by liquid-lysate method [35]. The purified phage was mixed with an activated culture of *A. baumannii* M13 (10^8^ CFU/ml) at MOI of 100, followed by shaking incubation at 180 rpm for 2 h at 37℃. The titer of the propagated lysate was counted using plaque assay.

For phage precipitation, polyethylene glycol precipitation (PEG) was performed following the next steps. First, 90 ml phage lysate with high titer (10^12^ PFU/ml) was prepared by liquid-lysate method. Second, 10% PEG-6000 and 1 M NaCL were added to 90 ml phage lysate, incubated on ice for 2 h, and kept overnight at 4℃. Third, the solution was centrifuged at 4800 × g, 4℃ for 30 min. The resulted PEG-precipitated phage was resuspended into 4 ml SM buffer, re-centrifuged, and the supernatant containing phage was filtered through 0.22 μm. Finally, an aliquot of the supernatant (phage precipitated with PEG) was directly subjected to transmission electron microscope (TEM), and the other aliquot was stored at −20℃ [36, 37].

### Phage morphology

Bacteriophage morphology was examined by TEM (Talos L20C G2, TEM, Thermofisher, Europe) at Damietta University (Damietta, Egypt). Phage lysate (Titer = 10^12^ PFU/ml) was fixed on a formvar carbon coated copper grid (Pelco International) using glutaraldehyde (2.5% V/V). Then, it was washed, negatively stained using 2% phosphotungstic acid (pH = 7), left to dry, and examined by TEM [38].

### Host range and efficiency of plating (EOP)

Host range of phage vB_AbaM_MU1 was established against a panel of seventeen strains of CRAB, Gram-negative (*E. coli*,* Klebsiella pneumonia*,* Pseudomonas aeruginosa*), and Gram-positive bacterial species (*Staphylococcus aureus*,* coagulative negative Staphylococcus aureus and Streptococcus sp.*). The activity of phage against each strain was determined using spot assay [33]. Sensitive strains were detected by lysis area after incubating overnight at 37℃.

The host range positive results were confirmed by efficiency of plating **(**EOP) [39]. Phage was serially diluted and spotted in triplicate over the bacterial lawn of each target strain and the host *A. baumannii* M13, followed by subsequent incubation for plaque counting. EOP was calculated as the ratio between the average PFU/ml count on target strain to the average PFU count on the host strain. The EOP was classified into four levels of efficiency as follows: EOP ≥ 0.5: high production; 0.1 ≤ EOP < 0.5: medium production; 0.001 < EOP < 0.1: low Production; EOP ≤ 0.001: inefficient.

### Adsorption profile

This experiment was conducted with minor modification [40]. The purified phage (titer = 10^11^ PFU/ml) was mixed with an exponentially grown culture of M13 (CFU/ml = 10^8^), achieving an MOI of 0.01. The mixture was incubated at 37℃ with shaking (180 rpm), and 1 ml was withdrawn at time intervals, centrifuged at 15.000 × g, 4℃, for 5 min. The supernatants were used for assaying the no. of free phages (unabsorbed phages) by plaque assay. The adsorption profile (%) was determined as: (initial phage titer PFU/ml – unabsorbed phage titer PFU/ml)/initial phage titer PFU/ml multiplied by 100%.

### Single step growth curve

Single step growth curve was demonstrated as a change in PFU/ml over time (min). Briefly, the purified phage (titer = 10^11^ PFU/ml) was mixed with 10 ml M13 culture (CFU/ml = 10^8^) at MOI = 0.001, and incubated for 12 min at 37℃ with shaking to facilitate adsorption. Then, it was centrifuged at 15.000 × g, 4℃ for 10 min, and the pellet was resuspended into 10 ml sterile nutrient broth medium. The suspension titer was immediately determined by plaque assay, 100 µl was withdrawn every 10 min over 110 min, and diluted to assess viral concentration (PFU/ml) at each time point. The latent period was calculated as the period after adsorption till releasing first phage progeny. The Brust size was determined as the ratio between the first released virions number at plateau phase (PFU/ml)/initial bacterial count (CFU/ml) [41].

### Phage stability

Thermal and pH stability of the purified phage was evaluated. For thermal stability, phage solution was incubated at a temperature range of (5℃ − 80℃) for 1 h, followed by counting of the infective plaques. For evaluating pH stability, 100 µl phage was incubated at 900 µl of different pH values (2–12) for 1 h, before immediate serial dilution in SM buffer. PFU/ml was counted, and incubated overnight at 37℃ [42].

The effect of ethanol was assayed against phage by spot-overlayer technique [43] with minor modification. Briefly, 100 µl of phage was mixed with 900 µl of various EtOH concentrations (10%, 50%, 75%, and 95%), and incubated for 15 min at room temperature. Then, phage titer was determined by serial dilution, spotting 5 µl over the bacterial lawn, and overnight incubation at 37℃.

### Time-killing curve

The phage was mixed with the M13 culture at different MOIs. First, 63 ml bacterial culture was prepared (10^8^ CFU/ml), and divided into 7 aliquots (9 ml each). Then, phage lysate (Initial PFU/ml = 10^12^) was serially diluted to obtain phage lysates with concentrations (10^10^, 10^9^, 10^8^, 10^7^, 10^6^, 10^5^ PFU/ml). Equal volume of phage lysate(s) was incubated with each of the seven *A. baumannii* M13 culture aliquots to obtain an MOI of 100, 10, 1, 0.1, 0.01, 0.001, respectively. An aliquot was left as a control by incubating equal volume of SM buffer with the bacterial host. Optical density (OD_600_) was measured at zero time, then the mixture density was measured every 30 min for 6 h. A line graph was plotted between time (min) and optical density for each MOI [44, 45].

### Phage DNA extraction, sequencing and bioinformatics analysis

High phage titer (10^10^ PFU/ml) was prepared, then DNA was extracted [46]. The extracted DNA was sequenced using Illumina MiSeq, followed by Illumina Nextera tagmentation pipeline (Illumina, Cambridge, UK) for the purpose of library preparation. Paired-end sequencing reads of 150 bp length were generated, then the quality of the reads was checked using FASTQC [47], and the poor-quality data were cleaned using PRINSEQ [48]. The trimmed data was subsequently assembled using SPAdes [49] at which multiple K-mers integrated to construct phage genome, and the quality of the assembled contigs were statistically assessed by QUAST [50]. Afterwards, open reading frames (ORF) were predicted by integrating ORF finder (Access date: July 2025; available at: www.ncbi.nlm.nih.gov/orffinder/). The coding sequences (CDSs) were manipulated using BLASTp [51] against NCBI Protein database (NR) to identify potential gene functions. Phage annotation was also conducted using PATRIC [52] and RASTtk [53] platforms, raising our fidelity in the deduced functions of genes. Subsequently, SNAP Gene was used to generate the corresponding genomic map, illustrating coding genes of assigned functions (GSL Biotech; access date: 18 July 2025; Available at: www.snapgene.com/). Phage vB_AbaM_MU1 has been submitted to GenBank with entry number PX093057. After that, PhageLeads tool was integrated to detect virulence, temperate genes or even antibiotic resistance genes [54]. The topology of the transmembrane among predicted proteins was further assessed using DeepTMHMM [55] since transmembrane proteins own a significant role in viral pathogenicity and replication process starting from genome releasing till generating viral progeny. Multiple approaches were followed to analyze vB_ABAM_MU1 phylogeny. BLASTn tool was used to identify highly similar phages to vB_AbaM_MU1 [51], considering VICTOR phylogeny for computing the genome-genome distance of top 100 matched phages in BLASTn [56]. Genome-BLAST Distance Phylogeny (GBDP) workflow was followed to conduct pairwise sequence alignments [57]. Moreover, VIRDIC workflow calculated intergenomic pairwise similarities with default parameters for species (> 95%) and genus (> 70%) thresholds [58]. The proteomic tree of vB_AbaM_MU1 was illustrated through Viral Proteomic Tree (ViPTree) [59] relying on tBLASTx [51] for computing genome-wide sequence similarities. CoreGenes 0.5 was further implemented to predict the orthologous (signature) genes between vB_AbaM_MU1 and the closely related phages [60]. Accordingly, phylogenetic tree of conserved proteins was generated by MEGA 12 [61] using top BLASTp matched proteins of other phages and CLUSTALW aligner with applying the default settings of fast adaptive bootstrap and best Maximum Likelihood fit model.

### Statistical analysis

All experiments were conducted in triplicate, as a form of technical replicates, and the results were illustrated in the form of mean ± standard deviation (SD). GraphPad Prism 8 software was used to generate all the graphs and perform all the statistical analyses. In this study, control(s) and treatment(s) data were statistically compared by one-way ANOVA, and Student’s t-test (two-tailed) at a significant level of *p* < 0.05.

## Results

### Susceptibility pattern

The sensitivity pattern of M13 was confirmed using Kirby-Bauer disk diffusion method, and the strain identity was confirmed as CRAB. It has shown resistance to all tested antibiotics: Ceftazidime (30 *µg*), Cefotaxime (30 *µg*), Piperacillin-tazobactam (100 *µg)*, Amikacin (30*µg*), Ampicillin-sulbactam (10*µg*), Ciprofloxacin (5 *µg*), Gentamicin (30 *µg*), Tetracycline (30*µg*), Levofloxacin (5 *µg*), Imipenem (10*µg*), Meropenem (*10 µg*), with being intrinsically resistant to Amoxicillin and Clavulanic acid (20/10*µg)*. The antibiotic sensitivity output of *A. baumannii* M13 was illustrated in the supplementary data (S2).

### Plaque and phage morphology

**Fig. 1:**
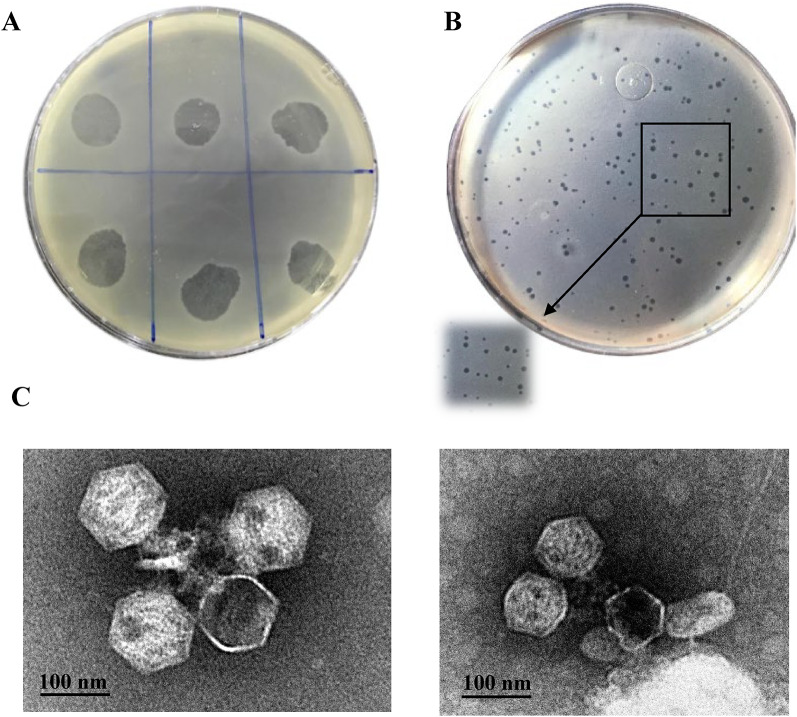
Cultural and Morphological characteristics of vB_AbaM_MU1. **(A)** Lytic activity of vB_AbaM_MU1 screened by spotting of filter-sterilized enriched sewage over soft layer of nutrient ager seeded with M13; (**B)** Purified plaques after plating vB_AbaM_MU1 higher dilutions at least three times over M13 lawn and incubating overnight at 37℃, the magnified part indicates small, identical and clear plaques; **(C)** TEM imaging of vB_AbaM_MU1, showing the [Left image] as phage particles morphology precipitated with PEG-6000 after incubating overnight at 4℃, while the [Right] showed the phage particle with a Myovirus morphotype. The scale bar is equivalent to 100 nm

After enriching the clarified sewage using *Acinetobacter* M13, spot overlayer analysis revealed clear spots on the host bacterial lawn, indicating the lytic potential of *A. baumannii* phage(s) (Fig. 1A). After several rounds of plaque purification, single ones were obtained showing spherical, clear and small plaques with 1 mm diameter over *A. baumannii* M13 lawn (Fig. 1B), the detected phage was labelled as vB_AbaM_MU1. Purification and propagation of phage vB_AbaM_MU1 yielded a high titer (10^12^ PFU/ml) which was used as a stock for the subsequent step.

After justifying phage vB_AbaM_MU1 under TEM, a hexagonal head with a contractile tail was observed (Fig. 1C). These viral features are distinctive characteristics of the Myoviral morphotype and categorized under Caudoviricetes class according to the International Committee on Taxonomy of Viruses (ICTV) [62].

### Host range determination with relative efficiency of plating (EOP)

**Fig. 2:**
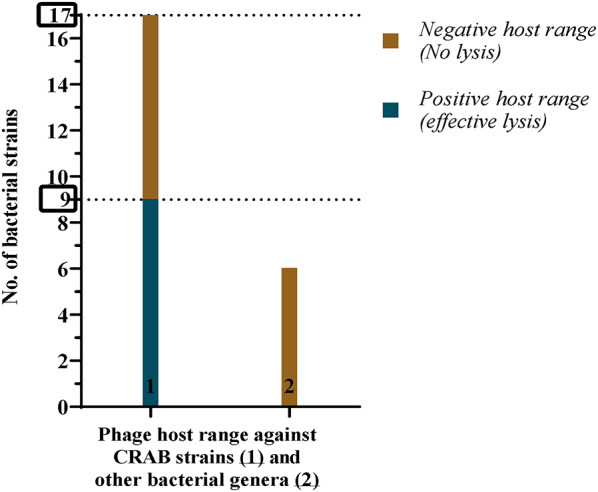
The phage showed activity against 9 of 17 CRAB strains (blue column indicates positive spot test)

Out of Seventeen CRAB, nine CRAB strains were recorded as sensitive to vB_AbaM_MU1 with different degrees of lysis. On contrast, vB_AbaM_MU1 was deemed ineffective against other tested bacterial genera such as *Klebsiella sp.*,* E. coli*,* Pseudomonas sp.*,* Streptococcus sp.*,* Coagulase-negative Staphylococcus aureus*,* and Staphylococcus* (Fig. 2).

vB_AbaM_MU1 was assessed in terms of EOP in Table 1 at which vB_AbaM_MU1 demonstrated an inefficient EOP (≤ 0.001) in all strains except one strain in which the phage yielded medium production (0.1 ≤ EOP < 0.5). Detailed data about host range strains are shown in the supplementary table S1. These findings indicate a relatively narrow host range phage with limited infection efficiency.

### Phage stability

**Fig. 3:**
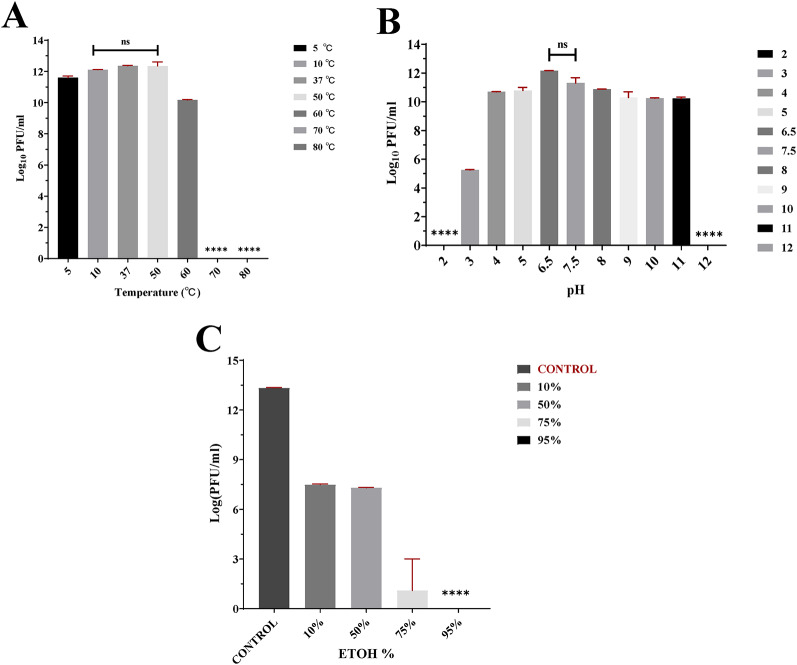
vB_AbaM_MU1 stability evaluation. **(A)** Bars of thermal stability at 50℃ compared to vB_AbaM_MU1 titer at other temperature degrees for 1 h incubation; **(B)** Bars of pH stability with Phage titer of pH at 6.5 compared to that of other pH range from 2 to 12 for 1 h incubation at 37℃; **(C)** Bars of different EtOH concentrations (10%, 50%, 75%, and 95%) with comparison of vB_AbaM_MU1 titer at given treatments to that of control after 15 min incubation at 25℃. The results were plotted as the Mean of three replicates with error bars of ±SD and statistically analyzed by t-test at a significance level of *P* < 0.05. Data classified as significant were represented by **** (*P* ≤ 0.0001), where ‘ns’ indicates statistically non-significant data (*P* > 0.05)

vB_AbaM_MU1 exhibited remarkable thermal tolerance, remaining stable at temperatures ranging from 5 °C to 60 °C. The phage titer decreased by 1 log at 5℃ (*p ≈* 0.01), whereas remained steady at 12 log_10_ PFU/mL at 10, 37, and 50℃. Although vB_AbaM_MU1 maintained its activity at 60℃, the phage titer was promptly reduced by 2 logs (*p ≈* 0.0001), comparable to that of 50℃. No activity was determined at 70 ℃ and above as explained in Fig. (3 A).

**Table 1:** EOP of vB_AbaM_MU1 against 9 CRAB *A. baumannii.* Productive infection was low for most isolates, with EOP ≤ 0.001 except for one strain

A. baumannii strains	EOP
8 1	EOP ≤ 0.001 (inefficient) EOP ≥ 0.1 (medium)

vB_AbaM_MU1 tolerated a wide range of pH (4.2–11) with the highest infectivity at pH of 6.5. At the acidic condition of pH 5 and below, the viral titer reduced by nearly 2 logs at pH 5 (*p ≈* 0.01), and 4 (*p* < 0.0001), respectively. A reduction of 7 log cycle at pH 3.2 (*p* < 0.0001) was determined, while no activity was detected at pH ≤ 2. At alkaline medium of pH 8 and above, a steady activity of 10 log_10_ PFU/mL was observed at pH of 8 (*p* < 0.0001), 9 (*p ≈* 0.02), 10 (*p ≈* 0.0001), and 11 (*p ≈* 0.001), followed by no activity at pH ≥ 12 as determined in Fig. (3B).

Turning to effect of ethanol, it was also shown to be virucide for vB_AbaM_MU1 **(**Fig. 3C**).** Phage titer treated with 10% and 50% ethanol was significantly dropped by 6 log cycle (*P* ≤ 0.0001) comparable to control. However, 10 log cycle reductions in titer treated with 75% ethanol (*p ≈* 0.0004) were justified till no observed activity in all dilutions at 95% alcohol.

### Phage adsorption profile and single step growth kinetics

**Fig. 4:**
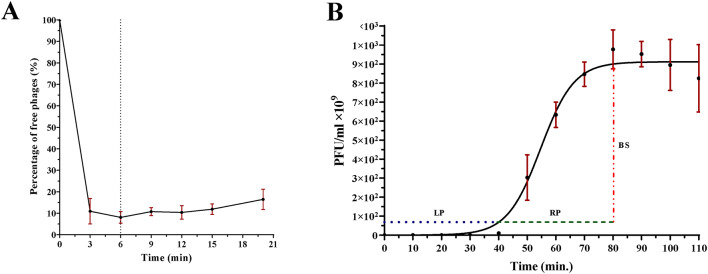
Biological characterization of vB_AbaM_MU1 (**A)** Phage Adsorption with shaking incubating conditions over a period of 20 min at 37℃. 92% of phage vB_AbaM_MU1 adsorbed within 6 min at MOI of 0.01 **(B)** Single step growth curve of Phage vB_AbaM_MU1 at MOI = 0.001 for 110 min with shaking incubation at 37℃. Latent period (LP) of ≈ 35–40 min (blue), lysis period (RP) of ≈ 40 min (green), and Burst size (BS) of 326 PFU/cell (red). The results were plotted as the Mean of three replicates with error bars of ± SD and statistically analyzed at a significance level of *P* < 0.05

Infection kinetics of vB_AbaM_MU1 were demonstrated using adsorption and one-step growth curve. Figure 4A at MOI = 0.01 declared that 89% of vB_AbaM_MU1 were adsorbed within 3 min, and approximately 92% were completely adsorbed within 6 min. No statistical differences were reported at all time intervals (*P* > 0.05).

From the observed kinetics of the given curve after vB_AbaM_MU1 replication at MOI of 0.001 (Fig. 4B**)**, the latent period approximately remained for 35–40 min, followed by lysis time of about 40 min, reaching the maximum count at 80 min with burst size near 326 PFU/infected cell. The data points given were statistically compared and deemed as significant (*P* ≈ 0.004).

### vB_AbaM_MU1 bacteriolytic curve

**Fig. 5:**
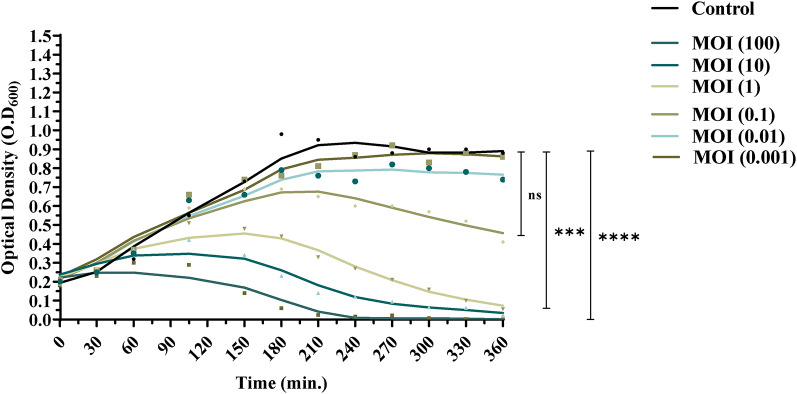
In-vitro bacteriolytic activity of vB_AbaM_MU1 at MOI (100, 10, 1, and 0.1, 0.01 and 0.001) after 6 h shaking incubation with M13 culture at 37℃. The results were plotted as the Mean of three replicates and statistically analyzed by t-test at a significant level of *P* < 0.05. Data classified as significant were represented by *** (*P* ≈ 0.0002), and **** (*P* ≤ 0.0001), where ‘ns’ indicates statistically non-significant data (*P* > 0.05)

Unlike the untreated M13 culture (control), a reduction in *A. baumannii* M13 concentration in the given treated group(s) was displayed and represented by a clear decline in the O.D reads at different MOI(s) of 100, 10, 1, 0.1, 0.01 and 0.001 as illustrated in Fig. (5). Variation in the O.D reads was determined after one hour-incubation from the start point of the experiment at all treatments except at MOI of 0.1. After 6 h incubation of both control and phage-treated culture(s), the O.D of the untreated culture increased from 0.2 to 0.8 ± 0.08. At MOI of 100, the O.D reached 0.00, reflecting the efficiency of killing by 100%. At MOI of 10, 1, 0.1, 0.01 and 0.001, the O.D recorded (0.02 ± 0.003), (0.05 ± 0.006), (0.4 ± 0.01), (0.7 ± 4), and (0.8 ± 6), indicating the lysis potential of phage vB_AbaM_MU1 by 97.3%, 93.6%, 53.4%, 15.9%, and 2.2%, respectively. Upon this lysis percentage(s), there is no significant bactericidal activity of phage vB_AbaM_MU1 at MOI of 0.1, 0.01 and 0.001. Data given at MOI 100, 10 (*p* ˂ 0.0001), and 1 (*p* ≈ 0.0002) were considered as statistically significant. No resistant bacterial strains were observed throughout the whole experiment, suggesting a high bacteriostatic efficiency of phage vB_AbaM_MU1 against the host M13 strain.

### DNA sequencing, genomic assembly and annotation of phage vB_AbaM_MU1

**Fig. 6:**
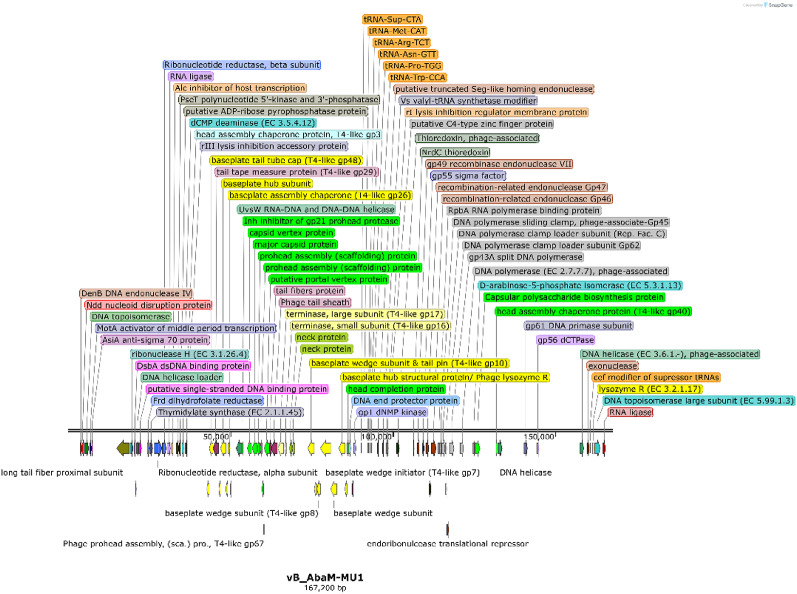
Genetic map of vB_AbaM_MU1 highlights coding sequences with assigned functions. Some functional genes were not illustrated due to space limits

**Fig. 7:**
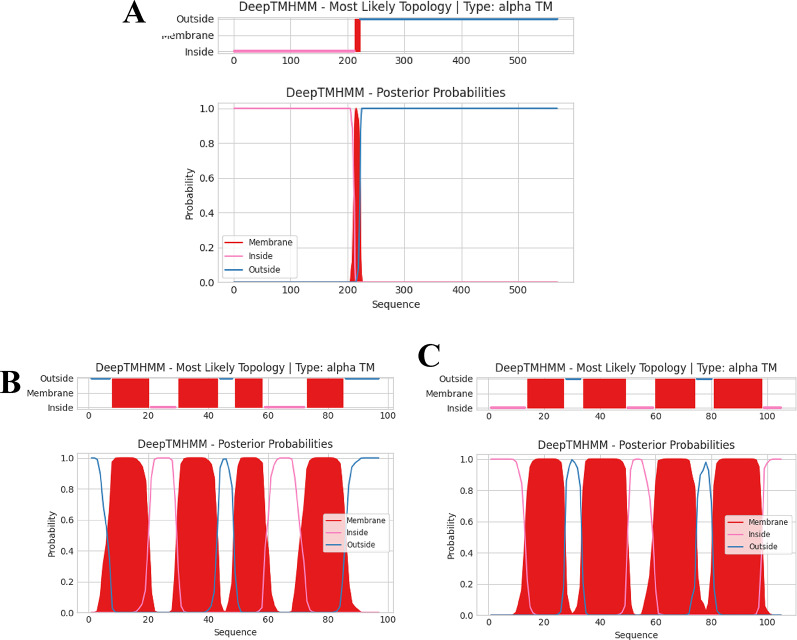
Transmembrane topology in **(A)** Phage tail tape measure protein (T4-like gp29) (ORF 56); **(B)** Hypothetical protein (ORF 167); **(C)** Hypothetical protein (ORF 203). X-axis: sequence position of amino acids; Y-axis: prediction probability; Red blocks: transmembrane domains; Blue line: domains outside the membrane; Pink line: domains inside the membrane

Sequencing of phage genome revealed that vB_AbaM_MU1 had a double-stranded DNA of ∼167, 200 bp in length, then the whole genome was submitted to GenBank under Acc. No. of PX093057. Furthermore, assembly demonstrated phage genome as a one contig with GC% 36.40; N50 167, 200; L50 1; and 0 N’s per 100 kb. After implementing multiple annotation platforms, two hundred and fifty-five protein-coding genes were predicted. Among these protein-coding genes, ninety-three were assigned to putative functions associated with DNA replication, transcription, packaging, and repair, along with phage structural, and cell lysis proteins (Supplementary Table S3). Phage MU1 had two hundred and fifteen OFRs on the plus strand, while forty OFRs are given on the complementary strand. Based on genomic analysis, six tRNAs were uncovered and entitled as Sup, Met, Arg, Asn, Pro, Trp with their anticodon sequences CTA, CAT, TCT, GTT, TGG, and CCA, respectively. Moreover, phage MU1 did not encode any lysogenic genes such as transposases or integrases. Extra genetic details were illustrated into the genetic map of phage MU1 (Fig. 6.), highlighting the coding sequences with assigned functions. Having screened phage MU1 genome by PhageLeads tool, no antibiotic resistance, bacterial virulence or even lysogenic-encoding lifecycle genes were detected, ensuring the safety and potential applicability of phage MU1 regarding therapeutic purposes. Concerning transmembrane domains (TMDs), twenty-three proteins with predicted TMDs were identified, derived from DeepTMHMM algorithm. Given the details of these TMDs, one TMD was predicted in ten proteins (OFRs: 2, 10, 56, 126, 156, 162, 190, 235, 237 and 239) with phage tail tape measure protein (T4-like gp29) of (ORF 56) as shown in Fig. 7A, followed by two TMDs in other ten proteins (ORFs: 6, 29, 103, 111, 122, 187, 215, 219, 230, and 233), while three TMDs were recorded in only one protein (ORF 216), along with four TMDs were detected in two proteins (ORFs: 167 and 203) as illustrated in Fig. 7B and C.

### Comparative phylogenetic analysis of phage vB_AbaM_MU1

**Fig. 8:**
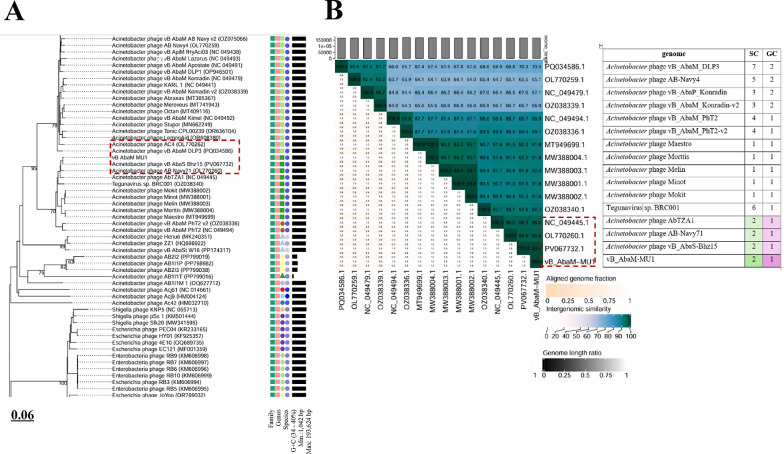
Phylogenetic analysis of vB_AbaM_MU1. (**A)** VICTOR phylogenetic tree of vB_AbaM_MU1 and top 80 BLASTn closely related phages; **(B)** VIRIDIC heatmap of phage vB_AbaM_MU1 and fifteen BLASTn closely related phages

**Fig. 9:**
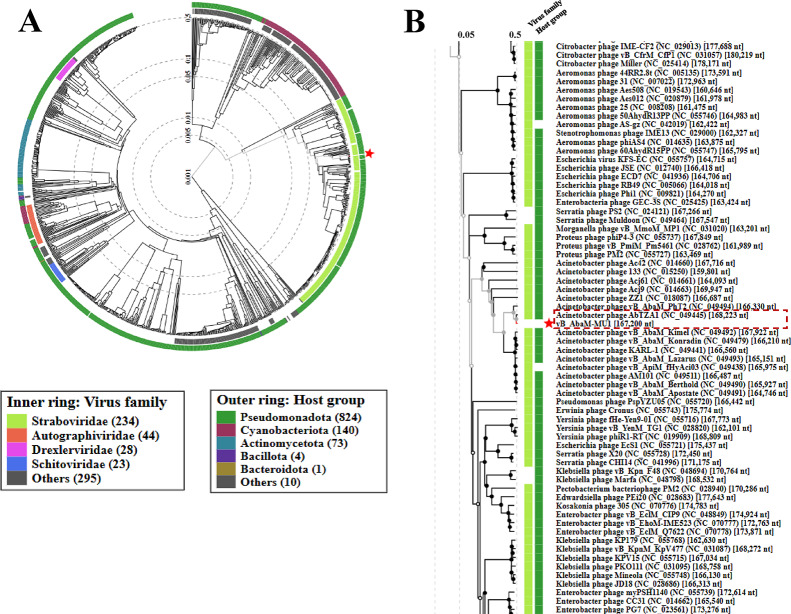
ViPTree proteomic tree of vB_AbaM_MU1 against RefSeq genomes of closely related phages. **(A)** Circular tree; **(B)** Rectangular tree illustrates a portion in the circular tree showing closely related phages

**Fig. 10:**
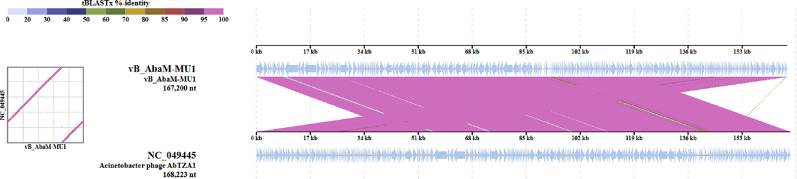
Complete genome comparison of vB_AbaM_MU1 and ViPTree closely related phage AbTZA1

**Fig. 11:**
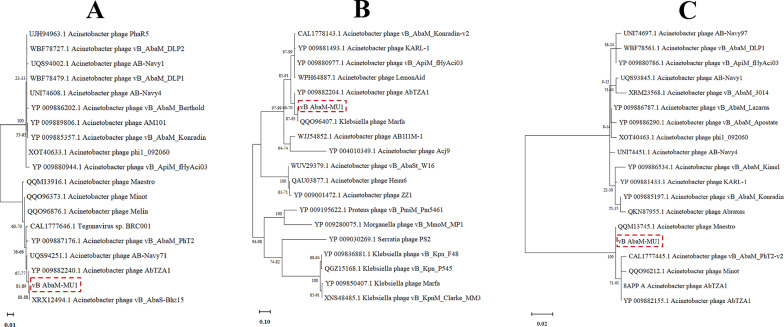
Phylogenetic analysis of the three signature proteins of vB_AbaM_MU1 and top matched BLASTp of other Phages using MEGA-12. **(A)** Phage sheath protein; **(B)** Capsid assembly protein; **(C)** Phage Endolysin (lysozyme R)

Based on VICTOR following phylogenomic GBDP platform analysis, phage vB_AbaM_MU1 phylogenetic tree had been derived from D6 formula which yielded 0% average support as explained in Fig. (8 A). OPT SIL clustering of VICTOR analysis generated twenty-eight species clusters, five clusters and one cluster over the generic and family levels, respectively. Phage vB_AbaM_MU1 had been positioned and aligned with closely genetically related *Acinetobacter* phages DLP3, Bhz15, AC4 and AB Navy71 as highlighted in the given VICTOR output figure. VIRDIC tool computed and highlighted the intergenomic similarities between phage vB_AbaM_MU1 and the top aligned BLASTn phages. *Acinetobacter* phage(s) vB_AbaS-Bhz15, AB-Navy71 and AbTZA1 were clustered within the same species and genus thresholds as vB_AbaM_MU1 with given similarity percentages 99.4%, 96.5% and 96%, respectively among the shortlisted 15 phages (Fig. 8B). Furthermore, VipTree output was visualized as two proteomic trees which confirmed that vB_AbaM_MU1 was a representative of the *Straboviridae* family, within *Caudoviricetes* class, and clustered with other *Acinetobacter* phages as illustrated in the circular and rectangular VipTree proteomic trees of Fig. (9 A, B). Referring to the most closely related phage(s) in the rectangular proteomic tree generated from VipTree, Phage vB_AbaM_MU1 was emphasized to be highly similar to *Acinetobacter* phage AbTZA1. Accordingly, the complete genome of Phage AbTZA1 was compared and aligned to that of vB_AbaM_MU1 to reveal the highlighted similarities and differences between both phages Fig. (10). After conducting coregenes analysis using these selected closely related *Acinetobacter* phages (AbTZA1, vB_AbaS-Bhz15, AB-Navy71, Melin and Maestro), two-hundred and thirty-nine orthologous genes were conserved between vB_AbaM_MU1 and these given phages based on their pan-genome analysis. Based on coregenes output, three signature protein-encoding genes (entitled as Sheath protein; Capsid assembly protein and Endolysin) were further opted for sequence alignment through BLASTp, following by visualizing the phylogenetic relationships with closely related proteins using MEGA-12. As observed in Fig. 11. (A-C), orthologues vB_AbaM_MU1 proteins were grouped and clustered with those of other *Acinetobacter* phages. Subsequently, phage vB_AbaM_MU1 was categorized under family *Straboviridae*, class *Caudoviricetes* based on the latest update of the International Committee on Taxonomy of Viruses (ICTV) [63].

## Discussion

In this study, we report on the isolation and characterization of a novel phage, vB_AbaM_MU1, targeting CRAB wound infection. The selected host strain used in our study (M13 strain) was previously classified as Extensively Drug Resistant (XDR). It carries about 25 important resistance genes, e.g. β-lactams (blaADC − 73, blaOXA − 23 (AbaR4b), blaTEM − 1 (AbGRI2-15)) and aminoglycosides (aphA1, armA, strA, strB) [29]. Previous studies [64, 65] have highlighted that broad host range phages are favorable in phage therapy and are analogous to wide spectrum antibiotics in phage treatment. Nevertheless, narrow host range phages could selectively be beneficial in certain infections since it minimizes off-target effects on beneficial microbiota while effectively combats resistant bacterial populations [66, 67]. In our study, vB_AbaM_MU1 demonstrated an effective infection in 9 out of 17 *A. baumannii* isolates, while showing no observed activity against other tested bacterial genera. This highlights its narrow host range specificity, a characteristic often noted in *A. baumannii* phages as indicated by other studies [68–71]. Despite this limitation, vB_AbaM_MU1 exhibited the ability to lyse multiple carbapenem-resistant *A. baumannii* (CRAB) strains.

Our findings revealed that vB_AbaM_MU1 retained its infectivity between 5 °C and 60 °C, indicating strong thermal tolerance that aligns with previous results for Abp95 reported in a previous study [72]. However, exposure to 70 °C and 80 °C resulted in complete loss of infectivity, like earlier reports for MRABP9 and Phab24, as they displayed similar heat sensitivity at these temperatures [70, 73]. In terms of pH stability, vB_AbaM_MU1 exhibited remarkable effectiveness across a wide acidic-to-alkaline range (pH 4.2 to 11), with optimal stability observed in a near-neutral habitat (pH 6.5–8). These results align with previously characterized *Acinetobacter* phages, including TCUP2199, P1068, vAbBal23, and vAbAbd25, which also exhibited a preference for neutral pH conditions [74–76]. However, most published *A. baumannii* phages exhibited stability at a wide range of pH, with peak stability being displayed at pH 7, vB_AbaM_MU1 showed optimal stability at pH 6.5 rather than 7, a deviation not commonly reported among other phages in this group. However, extreme conditions at pH 3 and 12 proved lethal, mirroring the pH sensitivity profiles of TCUP2199, P1068, Ф Ab4B, and MRABP9 [70, 74, 76, 77]. These results reinforce the resilience of vB_AbaM_MU1 in physiologically relevant conditions, making it a potential candidate for future research applications. Ethanol at high, moderate and even low concentrations was proved to be highly detrimental, causing a significant decline in phage titers. These results align with the data published by a previous study [78], in which the highest decline in phage titer was associated with ethanol. Despite the limited available number of reports investigating the influence of disinfectants on *A. baumannii* phages, our findings are consistent with recent studies [78–80]. These observations highlight the instability of vB_AbaM_MU1 in the presence of ethanol, emphasizing the importance of optimized storage conditions to maintain phage viability in therapeutic applications.

Regarding replication kinetics, 92% of phage vB_AbaM_MU1 was adsorbed within 6 min incubation on the host strain. Upon this, rapid adsorption within 5–10 min is a desirable characteristic of virulent phages [81], since adsorption is critical inevitable step, this action was reminiscent of three previously isolated phages: Acba_15 phage, TCUP2199 and, a temperate phage 5 W that had the same given features [74, 82, 83]. For more consideration, a short latent period between 35 and 40 min, and a high bursting (< 300 PFU/cell) were recorded in vB_AbaM_MU1. Although many *A. baumannii* phages with low to medium burst size have commonly been reported [42, 71, 72, 74, 82, 84, 85], the high burst size phages were confirmed in a few published *Acinetobacter* Caudoviricetes [73, 86], which are aligned with our phage vB_AbaM_MU1 as high bursting. Thus, vB_AbaM_MU1 phage is deemed as a promising candidate for future investigation. Bacteriolytic activity of phage vB_AbaM_MU1 was investigated at different multiplicity of infection (MOIs), and it was proved that the higher the MOI, the better the lysis with remarkable reduction in optical density (OD). The optimal MOI of phage vB_AbaM_MU1 was 1 since this ratio can effectively lyse the bacterial culture by 93.6% after 6 h of incubation with the host strain. Yet, it is worth noting that different methods were used for determining the optimal MOI. Thus, further investigation should be done to standardize the methodology for optimizing the decision of optimal MOI [86, 87]. Interestingly, no bacterial recovery was detected throughout the experiment. Therefore, these findings revealed the effective stability and antibacterial efficiency of the given phage. Our phage displayed an MOI-dependent reduction pattern roughly like TCUP2199 [74], and ABMM1 [42], but unlike growth characteristics from Phab24 [73], and vB_AbaM_P1 [86], as a MOI-independent reduction pattern.

Phage vB_AbaM_MU1 has been classified into the *Caudoviricetes* class, *Straboviridae* family based on sequencing data and morphological examination. vB_AbaM_MU1 has a head capsid and a contractile tail, classifying phage vB_AbaM_MU1 into T4-like Myovirus group with genomic size near 167 kbp. Both T4-like morphology and genome description are aligned with recent published research on *Straboviridae Acinetobacter* phages, entitled DLP1, DLP2, and vB_AbaSt_W16 that have anonymous genomic size of 164,355, 165,122, and 166,741 bp, respectively [23, 88]. The complete genome investigation facilitates the detailed characterization of new phages and addresses the gaps arising from experimental data. In silico examination of phage vB_AbaM_MU1 did not report any genes associated with antibiotic resistance, lysogeny or bacterial virulence. Thus, vB_AbaM_MU1 genomic analysis provides strong evidence for the safety and potential use as a therapeutic antimicrobial [27]. Identifying transmembrane regions in the candidate proteins could suggest potential roles of these proteins. DeepTMHMM tool identified multiple transmembrane domains, and the transmembrane topology prediction with the protein’s role, as it enables the passage of viral DNA into the infected bacterial cytoplasm by creating a channel in the bacterial cell membranes [89]. However, future studies on vB_AbaM_MU1 would focus on isolating and examining this potential protein activity. Various methodologies were applied for the phylogenetic analysis of vB_AbaM_MU1, and all these methods produced fairly similar results. VIRIDIC categorized vB_AbaM_MU1 alongside other *Acinetobacter* phages (vB_AbaS-Bhz15, AB-Navy71 and AbTZA). VIRIDIC phylogeny is highly reliable as it follows the ICTV guidelines in its algorithm [58]. In the same way, proteomic tree phylogeny indicated that vB_AbaM_MU1 is very similar to *Acinetobacter* phage AbTZA1. Proteomic trees provide insights into the evolutionary background of phages [90, 91], aiding in the exploration of more distant relationships. According to the recent update from the International Committee on Taxonomy of Viruses (ICTV), the host type and genomic traits (GC content, genome length, count of coding sequences) of the *Straboviridae* family, class *Caudoviricetes* [63], resemble those of phage vB_AbaM_MU1.

## Conclusion

CRAB remains a serious clinical challenge in hospital settings due to extensive drug resistance and the limited treatment options. In this study, the bacteriophage vB_AbaM_MU1 revealed lytic activity against the host CRAB isolate and demonstrated promising characteristics, including the absence of lysogenic genes, acceptable pH and thermal stability, and infection kinetics supportive of effective phage replication. Its sensitivity to high ethanol concentrations emphasizes the need for optimized handling and storage conditions. While MU1 displayed consistent in-vitro lysis and lacked identifiable virulence or resistance genes, these results reflect preliminary evidence only. vB_AbaM_MU1 should therefore be regarded as a promising research candidate, with future in-vivo studies needed to confirm its therapeutic potential prior to any clinical application. Further, future studies could explore phage cocktails or engineering strategies to broaden the host range of *Acinetobacter* phage MU1 while maintaining rapid adsorption and high bursting, which could improve the prospective applicability of this phage.

## Supplementary Information

Supplementary Material 1

Supplementary Material 2

Supplementary Material 3

## Data Availability

The dataset presented in this study can be found in online repositories. The nucleotide sequence of the *Acinetobacter* phage vB_AbaM_MU1 was deposited in NCBI GenBank database under accession number PX093057.
