# Supplementary material for: Phage vB_AbaM_MU1 for biocontrol of carbapenem-resistant Acinetobacter baumannii (CRAB) isolated from wound infection
Source: Virol J. 2026 Feb 6;23:49. doi: 10.1186/s12985-026-03066-9 (PMC12930573; doi:10.1186/s12985-026-03066-9)
Supplement: Supplementary file 1 — Supplementary Material 1 [file 12985_2026_3066_MOESM1_ESM.pdf]

Supplementary table S1

Isolate number	Accession number	origin	Spot test result	Lysis effect	EOP
**M1 (CRAB)**	(SAMN17265991)	Endotracheal tube	Negative	0	
**M2 (CRAB)**	(SAMN17265992)	Wound swab	Negative	0	
**M3 (CRAB)**	(SAMN17265993)	Blood	Positive	+3	0.001< EOP <0.1
**M4 (CRAB)**	(SAMN17265994)	Sputum	Positive	+3	0.001< EOP <0.1
**M5 (CRAB)**	(SAMN17265995)	Blood	Positive	+3	0.001< EOP <0.1
**M6 (CRAB)**	(SAMN17265996)	Sputum	Negative	0	
**M9 (CRAB)**	(SAMN17265999)	Blood	Negative	0	
**M10 (CRAB)**	(SAMN17266000)	Blood	Positive	+3	0.001< EOP <0.1
**M11 (CRAB)**	(SAMN17266001)	Pleural fluid	Negative	0	
**M12 (CRAB)**	(SAMN17266002)	Blood	Positive	+3	0.001< EOP <0.1
**M13 (CRAB)**	SAMN17266003	Wound swab	Host strain	+4	EOP ≥ 0.5
**M14 (CRAB)**	(SAMN17266004)	Urine	Negative	0	
**M15 (CRAB)**	(SAMN17266005)	Wound swab	Negative	0	
**M16 (CRAB)**	(SAMN17266006)	Blood	Negative	0	
**M17 (CRAB)**	(SAMN17266007)	Sputum	Positive	+3	0.001< EOP <0.1
**M18 (CRAB)**	(SAMN17266008)	Blood	Positive	+4	0.1≤ EOP <0.5
**M19 (CRAB)**	(SAMN17266009)	Blood	Positive	+3	0.001< EOP <0.1
**M20 (CRAB)**	(SAMN17266010)	Blood	Positive	+4	0.001< EOP <0.1
***Klebsiella sp.* (CRKP)**	Lab isolate	Blood	Negative	0	
***E. coli* (CREC)**	Lab isolate	Blood	Negative	0	
***Pseudomonas sp.* (CRPA)**	(SAMN42266025)	Blood	Negative	0	
Streptococcus sp.	Lab isolate	Blood	Negative	0	
Coagulase-negative Staphylococcus aureus	Lab isolate	Blood	Negative	0	
Staphylococcus aureus	Lab isolate	Blood	Negative	0	

CRAB: Carbapenem-Resistant *Acinetobacter baumannii*
CRKP: Carbapenem-resistant *Klebsiella pneumoniae*
CREC: Carbapenem-resistant *Escherichia coli*
CRPA: Carbapenem-resistant *Pseudomonas aeruginosa*

Supplementary table S1

The success of infection was assessed by a common system as follows (Kutter, 2009)[1]:
+4: Complete clearing.
+3: Clearing throughout but with fainty hazy background.
+2: Substantial turbidity throughout the cleared zone.
+1: A few individual plaques.
0: No clearing, but you may see a spot where the pipette tip touched the agar

EOP $\geq$ 0.5: **high production**
0.1$\leq$ EOP <0.5: **Medium production**
0.001< EOP <0.1: **Low Production**
EOP $\leq$ 0.001: **Inefficient.**

References:

[1.] Kutter E. Phage host range and efficiency of plating. Methods Mol Biol. 2009;501:141–9.
