# Supplementary material for: Phage vB_AbaM_MU1 for biocontrol of carbapenem-resistant Acinetobacter baumannii (CRAB) isolated from wound infection
Source: Virol J. 2026 Feb 6;23:49. doi: 10.1186/s12985-026-03066-9 (PMC12930573; doi:10.1186/s12985-026-03066-9)
Supplement: Supplementary file 2 — Supplementary Material 2 [file 12985_2026_3066_MOESM2_ESM.pdf]

Supplementary data (S2)

Antibiotic susceptibility profile of *A. baumannii* (M13) - SAMN17266003

A. baumannii (M13) was tested for its antibiotic susceptibility by using the disk diffusion method, and twelve commercial antibiotics discs were utilized. Based on the National Committee for Clinical Laboratory Standards Institute [1], the diameter of inhibition zone was interpreted, and the sensitivity pattern of M13 to the corresponding antibiotics were categorized as resistant, intermediate or sensitive as illustrated in **Table 1.** The response of M13 strain to different antibiotics classes was shown in **Figure 1,** and it was shown that M13 exhibited resistance (R) to the all 12 antibiotics-saturated discs, including Carbapenems, with different inhibition zone diameter (D_{mm}). Hence, *A. baumannii* (M13) was confirmed as Carbapenems-resistant *A. baumannii* (CRAB). Interpreted standards of inhibition zone diameter (D_{mm}) were explained in **Table 2.**

Table 1. Antibiotic susceptibility profile of *A. baumannii* (M13)

Antibiotic (s)	AMC 20/10µg	CAZ 30 µg	CTX 30 µg	PI 100µg	AK 30µg	A/S 10µg	CIP 5 µg	GEN 30 µg	TE 30µg	LEV-5 5 µg	IPM 10µg	MRP 10 µg
Zone diameter (D_{mm})	0mm	0mm	0mm	0mm	7mm	6mm	7mm	7mm	7mm	9mm	9mm	7 mm
Resistance pattern (R, I, and S)	R	R	R	R	R	R	R	R	R	R	R	R

NOTE: R: Resistant; I: intermediate; S: sensitive, and D_{mm}: Inhibition Zone Diameter in millimeter (mm).

**AMC:** Amoxicillin and Clavulanic acid	**AK:** Amikacin	**TE:** Tetracycline	**PI:** Piperacillin-tazobactam
**CAZ:** Ceftazidime	**A/S:** Ampicillin-sulbactam	**LEV-5:** Levofloxacin	**GEN:** Gentamicin
**CTX:** Cefotaxime	**CIP:** Ciprofloxacin	**IPM:** Imipenem	**MRP:** Meropenem

Supplementary data (S2)

Table 2. Interpreted standards of inhibition zone diameter (D_{mm}) for antibiotics susceptibility test concerning *Acinetobacter* sp. (CLSI, 2021)

**Antibiotic class**	**Antibiotic disk**	**Symbol**	**Diameter of inhibition zone (mm)**		
			**(S)**	**(I)**	**(R)**
Cephems	Ceftazidime (30 µg)	**CAZ-30**	≥ 18	15- 17	≤ 14
	Cefotaxime (30 µg)	**CTX-30**	≥ 23	15 - 22	≤ 14
β-Lactam combination agents	Piperacillin-tazobactam (100µg)	**PI-100**	≥ 21	18 - 20	≤ 17
	Ampicillin-sulbactam 10µg	**A/S-10**	≥ 15	12 - 14	≤ 11
	Amoxicillin and Clavulanic acid (20/10µg)	**AMC-20/10**	Intrinsically Resistant		
Carbapenems	Imipenem (10µg)	**IPM-10**	≥ 22	19 - 21	≤ 18
	Meropenem (10 µg)	**MRP-10**	≥ 18	15 - 17	≤ 14
Aminoglycosides	Amikacin (30µg)	**AK-30**	≥ 17	15 - 16	≤ 14
	Gentamicin (30 µg)	**GEN-30**	≥ 15	13 - 14	≤ 12
Tetracyclines	Tetracycline (30µg)	**TE-30**	≥ 15	12 - 14	≤ 11
Fluoroquinolones	Ciprofloxacin (5 µg)	**CIP-5**	≥ 21	16 - 20	≤ 15
	Levofloxacin (5 µg)	**LEV-5**	≥ 17	14 - 16	≤ 13

NOTE: R: Resistant; I: intermediate; S: sensitive. D_{mm}: diameter in millimeter

Supplementary data (S2)

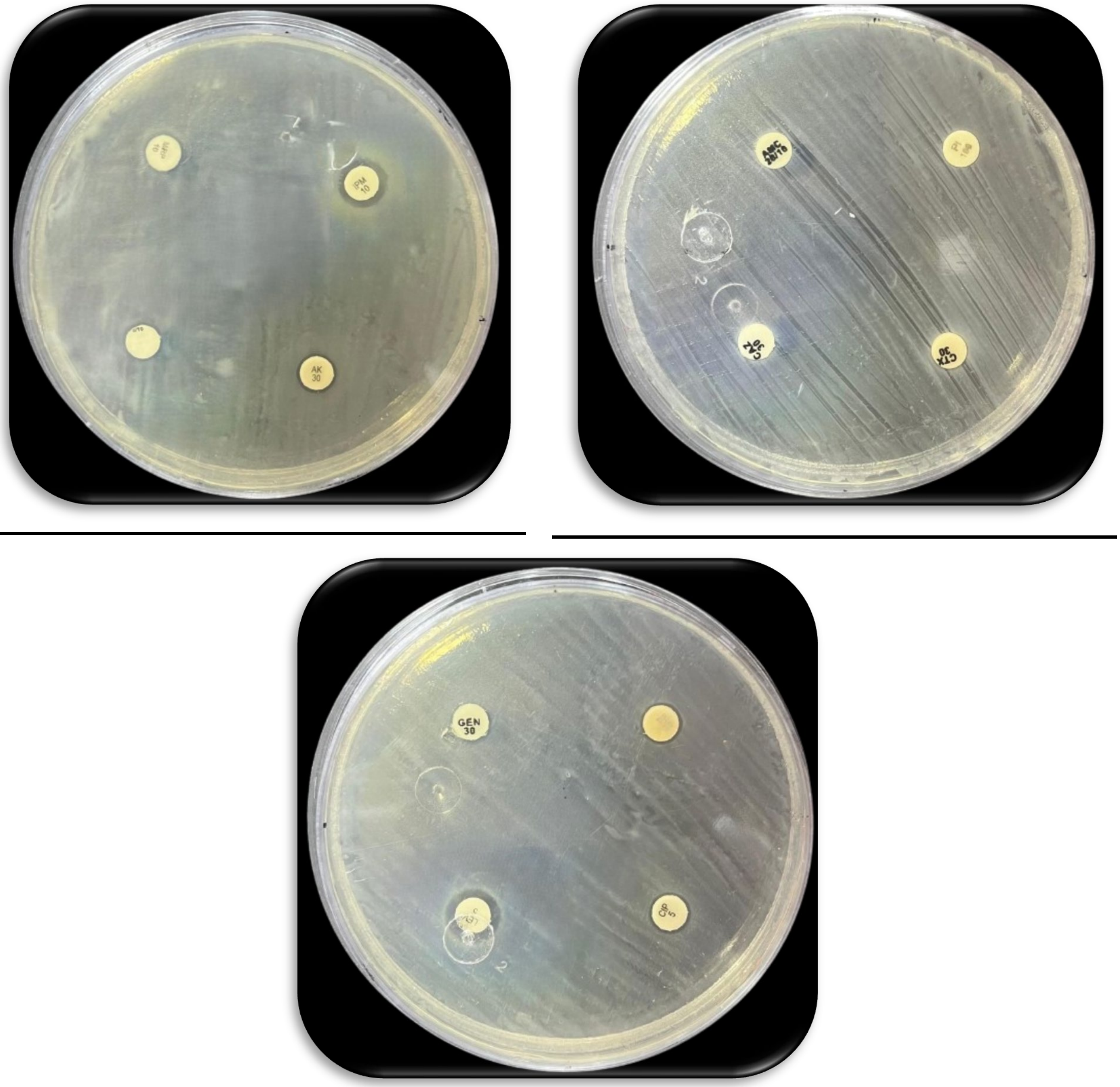

Figure 1. Muller-Hinton agar plates showing susceptibility pattern of *A. baumannii* (M13) - SAMN17266003 in response to antibiotics discs. *A. baumannii* (M13) exhibited resistance to all 12 tested antibiotics discs, including Carbapenems group. *A. baumannii* (M13) was confirmed as Carbapenems-resistant *A. baumannii* (CRAB).

Supplementary data (S2)

References:

1. M100Ed33| Performance Standards for Antimicrobial Susceptibility Testing., 33rd Edition. Clinical & Laboratory Standards Institute. [cited 2025 Feb. 25]. Available from: https://clsi.org/standards/products/microbiology/documents/m100/
